# A case of co-existence of muscle bridge and anomalous origin of coronary artery

**DOI:** 10.1016/j.ijscr.2019.02.049

**Published:** 2019-03-16

**Authors:** Federica Iezzi, Francesca Chiara Surace, Massimo Colaneri, Alessandro Capestro, Marco Pozzi

**Affiliations:** Department of Paediatric and Congenital Cardiac Surgery and Cardiology, Azienda Ospedaliero-Universitaria, Ospedali Riuniti Ancona “Umberto I, G.M. Lancisi, G. Salesi”, Ancona, Italy

**Keywords:** Coronary artery anomalies, Myocardial bridge, Case report

## Abstract

**Introduction:**

Coronary artery anomalies result from disturbances which may occur to the fetus in the third week of development. These disturbances may lead to anatomical variations which may affect the origin, trajectory, and termination of these arteries, or alterations in their intrinsic anatomy.

**Presentation of case:**

We report an unusual case in which both anomalous origin of the right coronary artery and myocardial bridge on left anterior descending artery were detected concurrently.

**Discussion:**

An investigation for myocardial bridge should be performed in all congenital coronary artery anomalies.

**Conclusion:**

Invasive testing could be considered in patients with myocardial and equivocal non-invasive functional testing, in order to obtain better risk stratification and treatment.

## Highlights

• Myocardial bridge is muscle overlying intramyocardial segment of an epicardial coronary artery.
• Arrhythmic complications were reported in patients with myocardial bridge.
• ARCA surgery is recommended in symptomatic patients.

## 1 Introduction

Anomalous origin of either the left main coronary artery or right coronary artery from the aorta and their course between the aorta and pulmonary trunk is rare with prevalence about 0,3–0,5% and mostly considered as a potentially fatal coronary artery anomaly [1].

Coronary anomalies are classified into four groups:

1) anomalies of coronary origin and trajectory (coronary artery absence, anomalous location of coronary ostium inside or outside the appropriate sinus of Valsalva, anomalous location of the coronary ostium in inappropriate sinus of Valsalva, and single coronary artery);
2) intrinsic abnormalities of coronary anatomy (atresia or stenosis of the coronary ostia, coronary aneurysm, coronary hypoplasia, and myocardial bridge);
3) anomalies of terminal coronary circulation (fistulas);
4) anomalous anastomotic vessels.

Some anomalies are only anatomic variants without clinical manifestations discovered accidentally or post mortem. Others can present with chest pain, syncope, or sudden cardiac death. Coronary angiography is the main diagnostic tool for almost of coronary artery diseases but computed tomography-angiography and cardiac magnetic resonance are non-invasive tests that give anatomical clarification about the origin and course of the coronary vessels in malformed vessels [2].

## 2 Case report

A 13-year-old young man was admitted to our hospital with non-sustained ventricular tachycardia episode, noticed during routine athletic evaluation.

**Fig. 1:**
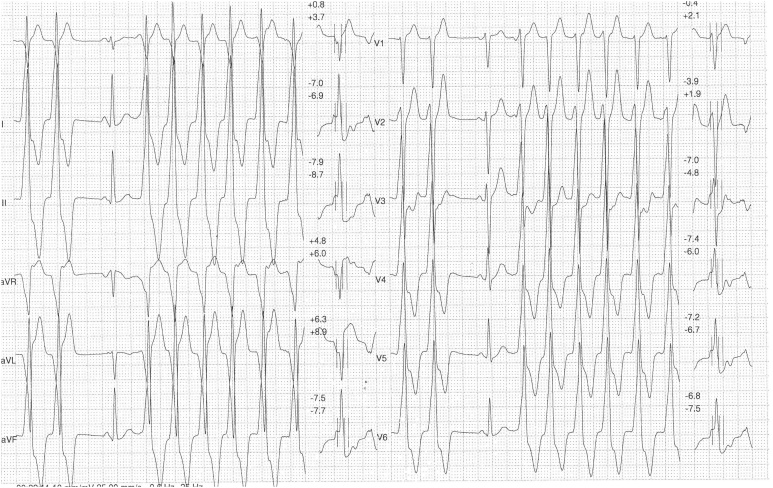
Non sustained ventricular tachycardia at exercise stress test (treadmill).

Resting ECG was normal, with sinus rhythm, normal heart rate in the absence of significant alterations of the ventricular repolarization phase (QTma × 413 ms, QTmin 383 ms, QTd 39 ms, QTcd 44 ms). Exercise stress test (treadmill) didn’t show signs of inducible ischemia through maximal effort (METS 21, HR max 194 beats per minutes), but induced asymptomatic non sustained ventricular tachycardia, with left bundle branch morphology an inferior axis with a rate of 150 beats per minute during the second minute of the recovery phase (Fig. 1).

Standard echocardiographic views showed a not clearly normal coronary pattern. Indeed, the right coronary artery appeared with high take-off from the aortic wall, without clear identification of right coronary artery ostium.

As a result of genetic screening for catecholaminergic tachycardia, beta-blocking therapy with nadolol was started and continued until the first cardiological follow up.

In order to exclude the presence of a possible coronary artery anomaly and disease, coronary computed tomography angiography was performed.

**Fig. 2:**
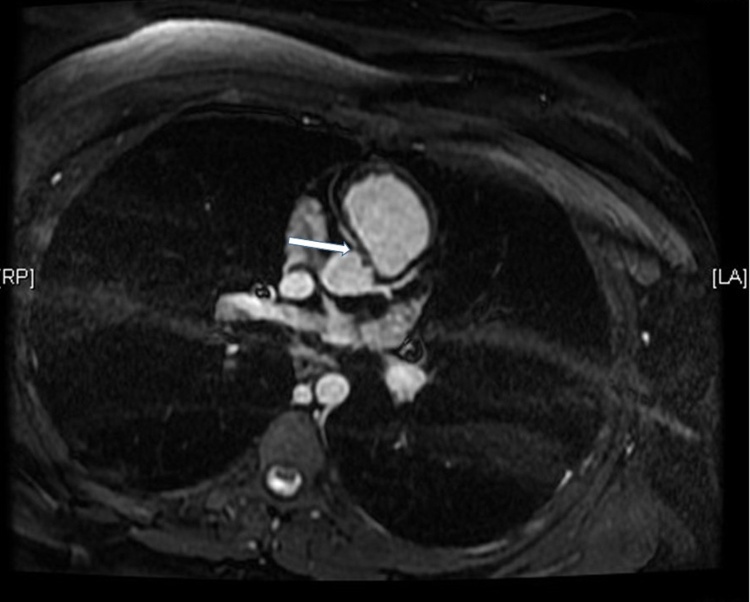
Cardiac magnetic resonance shows single coronary origin from left coronary sinus with malignant course of the right coronary artery (arrow).

The scan showed anomalous origin of the all three branches of coronary arteries of a single origin from left coronary sinus with malignant course of the right coronary artery, squeezed between the pulmonary trunk and the proximal ascending aorta. The distal part of the artery took its normal course. The left anterior descending artery and the circumflex artery calibers appeared to be normal. All the data were confirmed by cardiac magnetic resonance (Fig. 2).

**Fig. 3:**
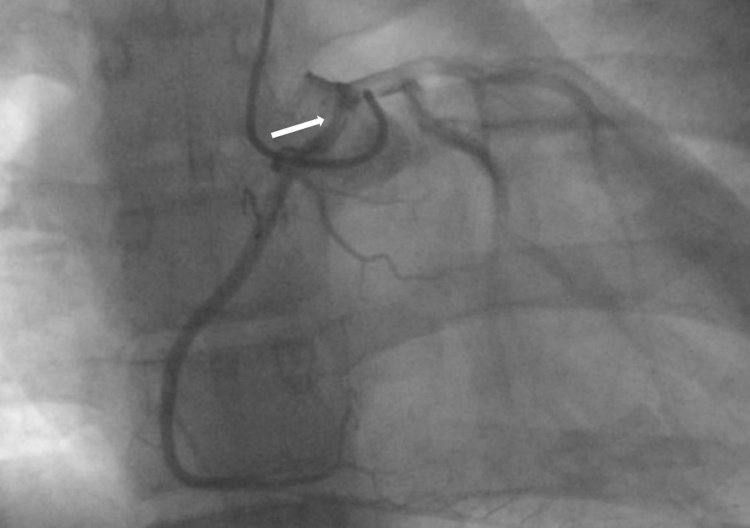
Catheter coronary angiography detects anomalous origin of coronary arteries from left coronary sinus, with slit like right coronary ostium (arrow).

Myocardial scintigraphy with protocol of two days steps and treadmill stress test (exercise) was performed, without significant evidences of perfusion defects. Catheter coronary angiography was performed to decide the tailored treatment plan. The coronary angiography showed the rare coronary anomaly pattern (Fig. 3).

Furthermore, the exam showed a significant milking effect at the middle segment of the left anterior descending artery, with borderline value of indices of intracoronary pressure and coronary flow reserve measured by Fractional Flow Reserve (FFR 0,74) and invasive Fractional Flow Reserve (iFFR 0,83) analysis.

On intravascular ultrasound (IVUS) both a slit like right coronary ostium and eccentric systolic compression in the proximal bridge segment of vessel were evident.

**Fig. 4:**
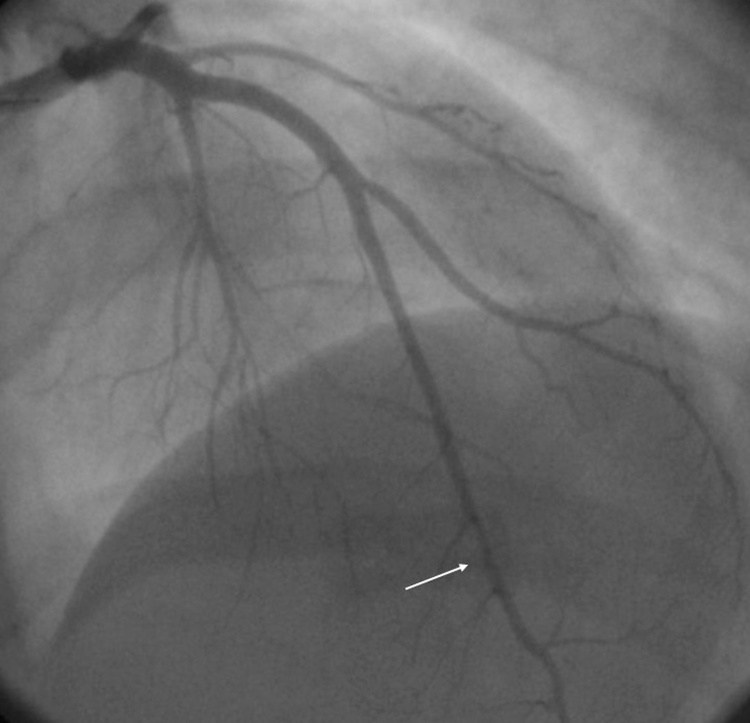
Catheter coronary angiography detects a bridging muscle in middle segment of the left anterior descending artery (arrow).

The depth and the length of the bridging muscle segment were measured as 16 mm and 25 mm, respectively (Fig. 4).

Planned on pump surgery was discussed. We performed a surgical unroofing of the right coronary artery intramural section and resuspension of the intercoronary commissure, that resulted in relocation of the coronary artery into the appropriate aortic sinus. Surgical myotomy involving resection of the overlying muscle fibers on middle segment of the left anterior descending artery was performed.

The patient's postoperative course was uneventful. He stayed overnight in the intensive care unit and left the hospital on postoperative day 7. No complications occurred during the first six months of follow-up.

## 3 Discussion

Anomalous origin of the right coronary artery (ARCA) from the left sinus of Valsalva and coursing between the aorta and pulmonary artery is a rare congenital abnormality with prevalence varying from 0.025% to 0.25% [3].

The treatment of ARCA is often controversial. Surgery is recommended in symptomatic patients and conservative management in asymptomatic patients. Reimplantation of the right coronary artery to aorta, unroofing of the ostium, osteoplasty, and bypass graft to distal right coronary artery with proximal ligation are the surgical options.

Muscle overlying the intramyocardial segment of an epicardial coronary artery, first mentioned by Reyman in 1737, is termed a myocardial bridge, and the artery coursing within the myocardium is called a tunneled artery. In 1960, Portman angiographically demonstrated occlusion of the coronary arteries with myocardial bridge in the systole. Myocardial bridge is most commonly localized in the middle segment of the left anterior descending artery.

Three treatment have been explored: negative inotropic and/or negative chronotropic agents (ie beta-blockers and calcium antagonists); surgical myotomy and stenting of the tunneled segment [4].

Myocardial perfusion via coronary arteries is damaged in systole in patients with myocardial bridge. This problem was related to consistent mechanical stress due to myocardial segment, coronary vasospasm in response to endothelial injury, and premature atherosclerosis. Intravascular ultrasound studies showed that coronary compression is also proceeding during diastole.

Several arrhythmic complications were reported in patients with myocardial bridge: sick sinus syndrome, paroxysmal atrioventricular block, exercise-induced ventricular tachycardia, and sudden cardiac death [5]. The relationship of myocardial bridge with arrhythmic events and ischemia was reported in small studies or case reports.

Prior studies in patients without myocardial bridge have suggested that QT dispersion reflects abnormal repolarization of the myocardium and an increased QT dispersion may predict ventricular arrhythmia [6].

Previous studies have reported a significant increase in QT dispersion and repolarization abnormalities during exercise in patients with myocardial bridge [7].

Neither nonsignificant stenosis proximal to the bridge nor systolic compression of the tunneled segment alone can sufficiently explain symptoms.

Occlusion mechanism, initially during systole only and then during continuing occlusion extending increasingly into diastole, resulted in distinct shortening of inflow time with significant reduction of epicardial flow, subendocardial flow, and distal coronary pressure.

An increase in sympathetic drive during stress or exercise likely facilitates ischemia, because tachycardia leads to an increase of the systolic-diastolic time ratio at the expense of diastolic flow. Increased contractility during stress further aggravates systolic (and diastolic) compression. Endothelial dysfunction and coronary artery spasm may also contribute to constriction of the tunneled segment.

The current gold standard for diagnosing myocardial bridges is coronary angiography with the typical ‘milking effect’ and a ‘step down-step up’ phenomenon induced by systolic compression of the tunneled segment. With the use of IVUS, and intracoronary pressure devices, morphological and functional features of myocardial bridging can be visualized and quantified. The ‘half-moon phenomenon’ is a characteristic IVUS observation [8].

The main question is whether an investigation for myocardial bridge should be performed in all congenital coronary artery anomalies or at least in coronary anomalies requiring surgery.

Flow alterations from this condition can cause accelerated atherosclerosis in the coronary segment immediately proximal to the bridged segment. The bridged portion itself is “spared” from atherosclerosis, likely through favorable shear forces resulting in increased expression of vasoactive agents as well as morphological changes in endothelial and smooth muscle cells in the area. Hemodynamic effects of bridging include systolic coronary flow reversal proximal to the bridge, as well as a decrease in coronary flow reserve. Clinical consequences range from angina to acute coronary syndrome to sudden cardiac death.

In the last decade, coronary computed tomography angiography has been introduced as an efficient, effective and safe method for evaluation of patients with atypical symptoms suggestive of coronary artery anomalies [9].

The evaluation of the vast majority of children with coronary artery anomalies and myocardial bridges is performed with non-invasive testing. However, a subset of these patients may benefit from invasive testing for risk stratification.

Assessment of anatomic details from within the coronary arteries using IVUS and physiologic testing of the significance of coronary artery lesions using FFR measurements, are routinely performed in adults with coronary artery disease. Instead most children may be followed based on clinical symptoms and non-invasive tests.

Due to the complex nature of some of these lesions, invasive testing, like coronary angiography, may be helpful in providing additional information that might be essential in the decision making process of their management.

Our limited experience shows that invasive testing may provide superior functional and anatomic data that are useful, especially in the setting of equivocal results of non-invasive testing.

Coronary angiography should be done in any case of diagnosed coronary artery anomalies to eventually detect associated myocardial bridge. Ostial morphology, interarterial or intramyocardial courses, and caliber changes of coronary arteries during the cardiac cycle can be delineated.

Based on our experience, invasive testing could be considered in patients with myocardial bridges that present with symptoms suggestive of ischemia, arrhythmia or with equivocal non-invasive functional testing, in order to obtain better risk stratification and treatment.

## Conflicts of interest

No conflict of interest.

## Sources of funding

No funding.

## Ethical approval

Ethical approval done from Ospedali Riuniti Ancona.

Consent form done by patient’s parent.

## Consent

Written informed consent was obtained from the patient for publication of this case report and accompanying images. A copy of the written consent is available for review by the Editor-in-Chief of this journal on request.

## Author contribution

Iezzi Federica developed the theory and performed the computations.

Surace Francesca Chiara developed the theory and performed the computations.

Colaneri Massimo verified the analytical methods.

Capestro Alessandro developed the theory and performed the computations.

Pozzi Marco supervised the findings of this work.

## Registration of research studies

researchregistry3300.

## Guarantor

Iezzi Federica.

## Provenance and peer review

Not commissioned, externally peer reviewed.

## References

[1] Angelini P. (2007). Coronary artery anomalies: an entity in search of an identity. Circulation.

[2] Agha R.A., Fowler A.J., Saetta A., Barai I., Rajmohan S., Orgill D.P., for the SCARE Group (2016). The SCARE statement: consensus-based surgical case report guidelines. Int. J. Surg..

[3] Alexander R.W., Griffiths G.C. (1956). Anomalies of the coronary arteries and their clinical significance. Circulation.

[4] Corban Michel T., Hung Olivia Y., Eshtehardi Parham, Rasoul-Arzrumly Emad, McDaniel Michael, Mekonnen Girum, Timmins Lucas H., Lutz Jerre, Guyton Robert A., Samady Habib (2014). Myocardial bridging: contemporary understanding of pathophysiology with implications for diagnostic and therapeutic strategies. J. Am. Coll. Cardiol..

[5] Erdogan H.I., Gul E.E., Gok H. (2012). Relationship between myocardial bridges and arrhythmic complications. J. Invasive Cardiol..

[6] Okin P.M., Devereux R.B., Howard B.V., Fabsitz R.R., Lee E.T., Welty T.K. (2000). Assessment of QT interval and QT dispersion for prediction of all-cause and cardiovascular mortality in American Indians: the Strong Heart Study. Circulation.

[7] Aksan G., Nar G., İnci S., Yanık A., Kılıçkesmez K.O., Aksoy O., Soylu K. (2015). Exercise-induced repolarization changes in patients with isolated myocardial bridging, medical science monitor. Int. Med. J. Exp. Clin. Res..

[8] Ge J., Jeremias A., Rupp A., Abels M., Baumgart D., Liu F., Haude M., Görge G., von Birgelen C., Sack S., Erbel R. (1999). New signs characteristic of myocardial bridging demonstrated by intracoronary ultrasound and Doppler. Eur. Heart J..

[9] Kim P.J., Hur G., Kim S.Y., Namgung J., Hong S.W., Kim Y.H. (2009). Frequency of myocardial bridges and dynamic compression of epicardial coronary arteries: a comparison between computed tomography and invasive coronary angiography. Circulation.

